# rs10514231 Leads to Breast Cancer Predisposition by Altering *ATP6AP1L* Gene Expression

**DOI:** 10.3390/cancers13153752

**Published:** 2021-07-26

**Authors:** Shumin Ma, Naixia Ren, Qilai Huang

**Affiliations:** Shandong Provincial Key Laboratory of Animal Cell and Developmental Biology, School of Life Sciences, Shandong University, Qingdao 266237, China; mashumin@sdu.edu.cn (S.M.); nch861896342@163.com (N.R.)

**Keywords:** risk SNP, breast cancer, gene regulation, *ATP6AP1L*

## Abstract

**Simple Summary:**

Breast cancer is the most common malignancy in women worldwide. Genome-wide association studies have identified thousands of genetic variants associated with predisposition to breast cancer. It is of vital importance to illustrate the biological mechanisms of these variants in breast cancer progression. Here, we revealed that rs10514231 affects breast cancer risk by altering *ATP6AP1L* expression through a functional interaction with TCF7L2. Our findings will be valuable for breast cancer risk prediction and effective targeted therapy for cancer patients.

**Abstract:**

Numerous genetic variants located in autophagy-related genes have been identified for association with various cancer risks, but the biological mechanisms underlying these associations remain largely unknown. Here we investigated their regulatory activity with a parallel reporter gene assay system in breast cancer cells and identified multiple regulatory SNP sites, including rs10514231. It was located in the second intron of *ATG10* and showed gene regulatory activity in most breast cancer cells we used. Mechanistically, the T allele of rs10514231 led to *ATP6AP1L* downregulation by decreasing the binding affinity of TCF7L2. Overexpression of the *ATP6AP1L* gene in cancer cells diminished cell proliferation, migration, and invasion. Notably, *ATP6AP1L* downregulation correlated with breast cancer risk and with poor prognosis in patients. These results provide a plausible mechanism behind the association of rs10514231 with breast cancer risk and will be important for more effective therapeutic target identification for precision medicine.

## 1. Introduction

Breast cancer is currently the most common malignant tumor among females worldwide, accounting for 24.5% of the new cancer cases and 15.5% of cancer deaths [1,2]. The disease aggregates in families and has an important inherited component. Meanwhile, genetic variants currently explain ~49% of familial breast cancer risk [3,4]. It is critical to understand the underlying genetic mechanisms leading to breast cancer. Genome-wide association studies (GWAS) have identified over 170 independent breast cancer susceptibility variants [5,6,7]. However, most SNPs reside within non-coding genomic regions, and there is little understanding of their target genes and the underlying regulatory mechanism [5].

Autophagy is a crucial catabolic process in which cytosolic cargo is engulfed by forming double-membrane autophagosomes and then degraded through fusing with lysosomes [8,9]. It is a highly conserved homeostatic process that mediates the turnover of intracellular protein aggregates and damaged organelles to maintain energetic homeostasis [10]. The biological role of autophagy in breast cancer is complex, and the current studies described this as a “double-edged sword” [11]. Cumulative evidence has reported that autophagy plays an essential role in breast cancer, which involved many *ATG*s (autophagy-related genes), such as *BECN1*, *ULK1*, *ATG5*, and *ATG10*, or autophagy signaling pathways, such as AMPK/mTOR [8]. Multiple variants in the autophagy gene regions have been reported for their association with cancer risk. However, the biological functions and underlying mechanisms are largely unknown. Most cancer risk SNPs possess gene regulatory roles and functions by altering gene expression. To identify regulatory SNPs, we previously developed a dinucleotide barcode-based parallel reporter gene assay system, DiR-seq [12]. We collected 22 autophagy gene-related cancer risk SNPs and evaluated their gene regulatory activity using the DiR reporter assay system in this work. We identified multiple regulatory SNP sites, among which the rs10514231 site showed significant regulatory activity in most breast cancer cell lines we used. Our further systematic mechanical study focusing on the rs10514231 site revealed that the variation affected *ATP6AP1L* gene expression by altering the chromatin binding of TCF7L2 to the therein regulatory element and led to breast cancer susceptibility.

## 2. Materials and Methods

### 2.1. Parallel Reporter Gene Assays Plasmid Library Preparation

We collected 22 cancer risk SNPs (5 SNPs from the GWAS catalog and 17 SNPs from publications) located in autophagy-related genes (Table S1). Then we got the surrounding sequences of the 22 cancer risk SNPs from the UCSC database on human genome assembly GRCh38/hg38. The 55 bp SNP-centered DNA fragments were ordered, annealed, and inserted upstream of promoter SV40 in the DiR reporter vectors digested with BglII (FD0083, Thermo Scientific, Waltham, MA, USA) and SmaI (FD0664, Thermo, Waltham, MA, USA). The ligation products were transformed into DH5α according to the manufacturer’s instructions (TSV-A07, Tsingke, Beijing, China). The next day, the single clones were picked and incubated in LB medium and grown at 37 °C for 12–16 h. The plasmid was extracted using a Plasmid Miniprep Kit (01519KA1, Axygen, Union City, CA, USA) and sent for Sanger sequencing with RVprimer3. Forty-four plasmids bearing both alleles of the 22 SNPs with a negative control plasmid were mixed equally and subjected to cell transfection as the DiR-seq reporter library. The related primers were listed in Table S2.

### 2.2. Cell Culture

All cell lines were originally purchased from the American Type Culture Collection (ATCC). 22Rv1, LNCaP, T-47D, ZR75-1, BT-549, and MCF7 cells were grown in RPMI-1640 (Invitrogen, Carlsbad, CA, USA); MDA-MB-468, MDA-MB-231, and Lenti-X 293T cells were grown in DMEM (Invitrogen); MDA-MB-453 and BT-474 cells were grown in DMEM/F12 (Invitrogen); and BT-20 cells were grown in EMEM (Invitrogen). A total of 10% FBS (Gibco, NYC, USA) and 1% antibiotics (penicillin and streptomycin, Sigma, St. Louis, MO, USA) were added to the basic medium. All the cells were cultured at 37 °C with 5% CO_2_.

### 2.3. Cell Transfection

The endotoxin-free plasmids prepared with the plasmid miniprep plus purification kit (DP01-Plus-300, GeneMark, Taiwan, China) were degermed by ethanol precipitation and subjected to transfection with FuGENE^®^HD Transfection Reagent (E2311, Promega, Fitchburg, WI, USA) according to the manufacturer’s instructions. Briefly, cells were seeded in the 6-well plate and transfection was performed on the next day when the cells reached approximately 70–90% confluence. For each transfection, 2 µg DNA and 6 µL transfection reagent was diluted in 150 µL Opti-MEM, separately, and then mixed together, followed by a 10 min incubation at room temperature. The mixtures were applied to cells gently, and the cells were put back in the a 37 °C incubator and grown for 24–48 h.

### 2.4. RNA Isolation and qRT-PCR Assays

Total RNA was extracted using the GeneJET RNA Purification Kit (K0732, Thermo) according to the manufacturer’s instructions. The mRNA was then treated with RapidOut DNA Removal Kit (K2981, Thermo) for 1 h at 37 °C, followed by DNaseI inactivation and RNA purification according to the kit protocol. Complementary DNAs (cDNAs) were synthesized using High-Capacity cDNA Reverse Transcription Kits (4374967, Applied Biosystems). For next-generation sequencing (NGS) library preparation, the sequence-specific primer named BarP6 (CACGATCTGTCCGCACTGCTTGG) was used for reverse transcription, and a random primer was used for other reverse transcription. The RT mixture was incubated at 25 °C for 10 min, followed by 120 min at 37 °C. Then, inactivated Transcriptor Reverse Transcriptase by heating to 85 °C for 5 min. RT-qPCR was performed with AceQ qPCR SYBR Green Master Mix (Vazyme) on the Rotor-Gene Q (Qiagen, Dusseldorf, Germany). The qPCR was performed following the program: 95 °C for 5 min of initial denaturation, then 40 cycles of 95 °C for 30 s, 60 °C for 30 s, and 72 °C for 10 s with fluorescence acquirement, followed by a final melting curve step. The relative expression of the target genes was determined using the comparative Ct method and normalized with the reference gene actin beta using the primers as listed in Table S3.

### 2.5. DiR-seq Library Construction and Sequencing

The cDNA products were amplified in two rounds of PCR for DiR-seq library construction using 2× Phusion Hot Start II High-Fidelity PCR Master Mix (F565L, Thermo Scientific, MA, USA). In the first round of PCR, the binding sites of the Illumina sequencing primers were added at both ends. In the second round of PCR, adaptors for cluster generation and the index sequences were added. The first-round PCR was performed using the program: 98 °C for 30 s of initial denaturation, then 7 cycles of 98 °C for 10 s, 72 °C for 45 s, and followed by a final extension at 72 °C for 5 min. The PCR products were purified using 1 × VAHTS DNA Clean Beads (N411, Vazyme, Nanjing, China). The second-round PCR was performed with 1 ng template DNA using the cycling program: 98 °C for 30 s of initial denaturation, then 10 cycles of 98 °C for 10 s, 72 °C for 45 s, and followed by a final extension at 72 °C for 5 min. The PCR products were purified and eluted in 1 × TE buffer. As input control, we also subjected the plasmid library for the above two rounds of PCR. The final purified libraries were quantified with a Nano-Drop 300 and then sent for paired-end sequencing on the Illumina HiSeq X-TEN platform run by GENEWIZ (Suzhou, China).

### 2.6. DiR-seq Data Analysis

We used the software FastP (https://github.com/OpenGene/fastp, accessed on 3 December 2018) to process the Illumina sequencing raw data. The paired reads were assembled using the software Panda-seq, and the sub-libraries were sorted out using the R package ‘ShortRead’. Next, the reads of each dinucleotide barcode were counted with the R package ‘ShortRead’. The barcode counts were normalized with the corresponding barcode counts from the plasmid constructs to represent the reporter expression level for a given SNP allele. The regulatory activity of the negative control was normalized as 1. We defined the regulatory SNPs where the reporter expression level was significantly different (*p* < 0.05) between the protective and risk allele, and the regulatory activity fold change of risk/protective was either <0.8 or >1.2.

### 2.7. Formaldehyde-Assisted Isolation of Regulatory Element Assay (FAIRE)

A FAIRE assay was conducted as previously described [13]. Cells were fixed with 1% formaldehyde for 10 min at room temperature and quenched using 125 mM glycine for 5 min. Then cells were collected, washed twice with cold PBS, and could be stored at −80 °C for subsequent use. Alternatively, the cell pellet was resuspended in lysis buffer (20 mM Tris-HCl, pH 8.0, with 10 mM KCl, 10% glycerol, and 2 mM DTT) supplied with 1× cOmplete Protease Inhibitor (Roche Diagnostics, Basel, Switzerland) and rotated at 4 °C for 30 min. After being centrifuged at 5000× *g* for 5 min, the cell nuclei were collected, resuspended in 2% SDS lysis buffer (50 mM Tris-HCl, pH 8.1, with 2% SDS, 10 mM EDTA, and cOmplete Protease Inhibitor), and rotated for 30–60 min at 4 °C. Samples were then sonicated with a Bioruptor Pico (Diagenode, Seraing, Belgium) to an average size of about 200 bp. Cellular debris was cleared by centrifugation at 13,000× *g* for 5 min at 4 °C. The chromatin lysate containing 500 ng DNA was extracted twice with phenol/chloroform/isoamyl alcohol (Invitrogen) and then once with chloroform/isoamyl alcohol. DNA in the aqueous phase was then precipitated with ethanol in the presence of 20 µg of glycogen (Thermo Scientific) and resuspended in 10 mM Tris-HCl (pH 7.4). A 1/10 volume of the chromatin lysate was taken as the control. The FAIRE DNA and control were subjected to overnight reverse cross-linking at 65 °C. Then the DNA was purified using 1 × VAHTS DNA Clean Beads (N411, Vazyme), and finally analyzed by qPCR detection with the primers listed in Table S3. The qPCR was performed following the program: 95 °C for 5 min of initial denaturation, then 40 cycles of 95 °C for 30 s, 60 °C for 30 s, and 72 °C for 10 s with fluorescence acquirement, followed by a final melting curve step.

### 2.8. Chromatin Immunoprecipitation (ChIP)

A ChIP assay was performed as previously described [14]. Briefly, cells were fixed, lyzed, sonicated, and chromatin lysate was prepared as described in the FAIRE experiment. A total of 30 μL of Magna ChIP™ Protein A + G Magnetic Beads slurry (16-663, Merck) for each reaction was washed twice with blocking buffer (0.5% BSA in IP buffer (20 mM Tris-HCl, pH 8.0, with 2 mM EDTA, 150 mM NaCl, 1% Triton X-100 and Protease inhibitor cocktail)), followed by 12 h incubation at 4 °C with 5 μg of Monoclonal ANTI-FLAG^®^ M2 antibody (F1804-50UG, Sigma-Aldrich, St. Louis, MO, USA) or normal mouse IgG (sc-2025, Santa Cruz Biotechnology) in 1 mL blocking buffer. Then the complexes were washed twice with blocking buffer and incubated with diluted chromatin lysate (about 30 μg) in IP buffer (supplied with 0.2% SDS, 75 mM NaCl, 0.5% Triton-100) for 12 h. Next, the complex was washed twice in turn with wash buffer I (20 mM Tris-HCl, pH 8.0, with 2 mM EDTA, 0.1% SDS, 1% Triton X-100, and 150 mM NaCl), buffer II (20 mM Tris-HCl, pH 8.0, with 2 mM EDTA, 0.1% SDS, 1% Triton X-100, and 500 mM NaCl), buffer III (10 mM Tris-HCl, pH 8.0, with 1 mM EDTA, 250 mM lithium chloride, 1% deoxycholate, and 1% NP-40), and buffer IV (10 mM Tris-HCl, pH 8.0, and 1 mM EDTA). Finally, 50 μL of extraction buffer (10 mM Tris-HCl, pH 8.0, 1 mM EDTA, and 1% SDS) was added to extract the DNA-protein complexes from the beads. Then the DNA-protein complex was incubated with RNaseA for 30 min at 37 °C and subjected to reverse cross-linking. The DNA was purified using 1 × VAHTS DNA Clean Beads, and the target DNA fragments were analyzed by qPCR. The primers for the ChIP-qPCR are listed in Table S3. The qPCR was performed following the program: 95 °C for 5 min of initial denaturation, then 40 cycles of 95 °C for 30 s, 60 °C for 30 s, and 72 °C for 10 s with fluorescence acquirement, followed by a final melting curve step.

### 2.9. Lentivirus Production and Infection

The lentiviral vectors were packaged using the third-generation system in the Lenti-X 293T cells. Briefly, the Lenti-X 293T cells were seeded in 6-well plates and grown for 12–24 h to reach 70–90% confluence. Then the growth medium (DMEM with 10% FBS and 1% antibiotics) was replaced with 1 mL pre-warmed DMEM without FBS and antibiotics, and the cells were co-transfected with the lentiviral transfer vector (1.5 μg), pVSVG (envelope plasmid, 0.5 μg), pMDLg/pRRE (packaging plasmid, 0.5 μg), and pRSV-Rev (packaging plasmid, 0.5 μg) plasmids using 4.5 μL PEI (408727, Sigma). After 4–8 h, we changed the medium with fresh DMEM containing 10% FBS (Gibco) and 1% antibiotics (penicillin and streptomycin). A total of 24 h later, the virus-containing medium was collected every 12 h up to six times. The virus supernatant was centrifuged for 5 min at 1000 rpm and passed through a 0.45 μm filter unit (Millipore, MA, USA), then stored at −80 °C or used directly for subsequent experiments.

Before viral infection, the host cells were seeded in a 6-well plate and grown for 16–24 h to reach a density of 60–70%. Then, the growth medium was replaced with the virus supernatant containing 8 μg/mL polybrene (Sigma). A total of 24 h later, the medium containing virus was removed and replaced by a normal medium containing puromycin (Sigma) (2 μg/mL for MDA-MB-453, 1 μg/mL for MDA-MB-468 cells). When the control cells without virus infection were all dead, the cells were cultured in a normal growth medium. After 2 days, the target cells were collected for subsequent experiments.

### 2.10. Gene Knockdown and Overexpression

The shRNA sequence (TRCN0000262847 and TRCN0000262848) targeting *TCF7L2* was designed according to MISSION^®^ shRNA Plasmid DNA (MERCK, Branchburg, NJ, USA) and inserted into the pLKO.1 vector. The *TCF7L2* and *ATP6AP1L* CDS sequences were amplified from the T-47D cDNA and inserted into the lentiCRISPR v2 (Addgene plasmid #52961) between AgeI and BamHI. The PCR was performed following the program: 95 °C for 5 min of initial denaturation, then 35 cycles of 95 °C for 10 s, 60 °C for 10 s, and 72 °C for 30 s, and finally 72 °C for 5 min. Then the lentivirus was packaged and infected with breast cancer cells as per the procedure mentioned above. The gene expression level was measured by qPCR. The qPCR was performed with the program: 95 °C for 5 min of initial denaturation, then 40 cycles of 95 °C for 30 s, 60 °C for 30 s, and 72 °C for 10 s with fluorescence acquirement, followed by a final melting curve step.

### 2.11. Genome Editing Using CRISPR/Cas9

The gRNA sequence targeting rs10514231 was designed based on the NGG protospacer adjacent motif (PAM) of *S. pyogenes* Cas9 [15]. The oligos were annealed and ligated with the BbsI digested vector pSpCas9 (BB)-2A-Puro (PX459) V2.0 (Addgene plasmid #62988). The resulting plasmids were then transfected into MDA-MB-453 cells using FuGENE^®^HD Transfection Reagent (E2311, Promega, Madison, WI, USA) according to the technical manual. The medium was changed with the fresh medium containing 2 μg/mL puromycin 24 h later. After the non-transfected cells died completely, the survived transfected cells were passaged, with a part used for the editing efficiency evaluation using the getPCR method, as described previously [16]. Single-cell clones were isolated using the limited dilution method in 96-well plates, and the genotypes were determined by the getPCR assay and confirmed through Sanger sequencing. The primers for the getPCR analysis are listed in Table S3. The getPCR was performed using the program: 95 °C for 5 min of initial denaturation, then 40 cycles of 95 °C for 30 s, 69 °C for 30 s, and 72 °C for 10 s with fluorescence acquirement, followed by a final melting curve step.

### 2.12. Cell Viability and Proliferation Assays

The MDA-MB-453, MDA-MB-468, and T47D cells that underwent lentivirus infection were trypsinized, resuspended, and seeded into 96-well plates at a density of 8 × 10^3^ per well. Cell viability and proliferation were measured with CCK-8 (MA0218, Meilun, Dalian, China) at designed time points by reading the absorbance at 450 nm, following the manufacturer’s instructions. Values were obtained from three independent replicate wells, and the statistical significance was calculated using a two-tailed Student’s *t*-test.

### 2.13. Wound Healing Assays

Cells were seeded into a 6-well plate and cultured to reach 100% confluence. The monolayers were scratched using a sterile 200 μL pipette tip and washed twice with PBS to remove the cell debris and then supplied with the fresh medium containing 5% FBS. The wound areas were imaged at 24 h intervals using an inverted fluorescence microscope (IX53, OLYMPUS). The area of the wound in each well was analyzed using the Image J software [17].

### 2.14. Colony-Forming Assay

Cells were seeded into 6-well plates at a density of 2000 per well and grown at 37 °C with 5% CO_2_, with fresh medium replaced gently every three days. Then, 15–20 days later, the medium was removed, and the cells were washed twice with 1XPBS carefully without disturbing a single colony. Then cells were fixed with 100% methanol for 15 min and then stained with 0.05% crystal violet staining solution (HY-B0324A, MCE) for 10 min. Finally, cells were washed with deionized water, dried overnight, and photographed. Quantitative changes in clonogenicity were determined by extracting the colonies with 1% SDS in 0.2 N NaOH for 1 h and measured the absorbance at 570 nm. The values were from three biological replications, and the statistical significance was calculated with a two-tailed Student’s *t*-test.

### 2.15. Transcription Factor Prediction

We used the HaploReg v4.1 tool online (https://pubs.broadinstitute.org/mammals/haploreg/haploreg.php, accessed on 1 May 2019), and JASPAR 2016 (http://jaspar2016.genereg.net/, accessed on 1 May 2019) for transcription factor prediction. For the HaploReg v4.1 tool, we entered the SNP rs10514231 in the query space and directly got detailed information about the rs10514231 site, such as the regulatory chromatin states DNase-seq and histone ChIP-Seq, regulatory motifs altered, and so on. At the same time, we also predicted the transcription factor on JASPAR 2016 online by entering the FASTA sequence of 21 bp SNP-centered DNA in the SCAN interface on the Browse JASPAR CORE Vertebrata page and selected all the transcription factors for analysis with a 90% score threshold. TCF7L2 was the predicted transcription factor in both of these tools. 

### 2.16. Statistical Analysis

The two-tailed Student’s *t*-test was applied to assess the statistical significance for differential analysis as indicated in the corresponding sections. For the association analysis between the rs10514231 genotypes and gene expression levels, we used the online Genotype-Tissue Expression (GTEx) Portal v8 (or dbGaP accession phs000424.v8.p2) database (https://gtexportal.org/home/, accessed on 20 October 2020). By simply inputting the SNP ID and then choosing the tissue type, we could obtain the violin plot of the potential eQTL genes in a given tissue. The statistical significance was assessed using two-tailed two-sample *t*-tests with Welch correction. 

The Kaplan–Meier survival analysis was performed on the Kaplan–Meier plotter online tool (http://kmplot.com/analysis/, accessed on 25 June 2021) [18], in which the gene expression data, relapse-free, and overall survival information were originated from the GEO, EGA, and TCGA databases. We split patients according to the *ATP6AP1L* expression level by choosing the “auto select best cutoff”, and the survival analysis for the cancer subtypes was performed by restricting analysis with Subtype-PAM50. The Kaplan-Meier survival plots were generated, and the hazard ratio with 95% confidence intervals and log-rank *p* value were calculated.

We downloaded transcriptome data (project ID: TCGA-BRCA, dbGaP Study Accession: phs000178) from TCGA (https://portal.gdc.cancer.gov/, accessed on 29 March 2019) using the R package “TCGAbiolinks”. The gene annotation file GDC.h38 GENCODE v22 GTF was used to match the data file to the TCGA ID and subgroup analysis was performed with the breast cancer subtype file PAM50 (only for breast cancer) [19]. The R package “Deseq2” was used to process the count data for the transcriptome. The “Deseq2” normalized data could be used to analyze the gene expression difference between groups. The R package “GGplot2” was used to visualize the data with a box plot. The Mann–Whitney *U* test was used to evaluate the statistical significance for differential gene expression analysis in normal tissues and breast cancer tissues.

## 3. Results

### 3.1. Parallel Reporter Gene Assays Identify Regulatory SNPs

We collected 22 cancer risk SNPs (Table S1) located in autophagy-related genes from the GWAS catalog and publications to evaluate their potential gene regulatory activity with the DiR system. As previously described [12], we synthesized the 55 bp oligos centered on the SNP for both alleles of the 22 risk SNPs and inserted them into the DiR barcode vectors upstream of the SV40 promoter. In the resulted reporter construct library, one SNP allele corresponds to one given barcode in the transcribed reporter region. The constructs were then transfected into nine breast cancer cell lines and two prostate cancer cell lines for DiR-seq assay. The prostate cancer cell lines were used for comparison to investigate if the regulatory function of the risk SNP is specific in breast cancer cells. The RNA was isolated, reverse transcribed, and then the barcode region was amplified to construct the next-generation sequencing library. The barcode counts from the cDNA samples were normalized with the corresponding barcode counts from the plasmid constructs to represent the reporter expression level for a given SNP allele.

**Figure 1:**
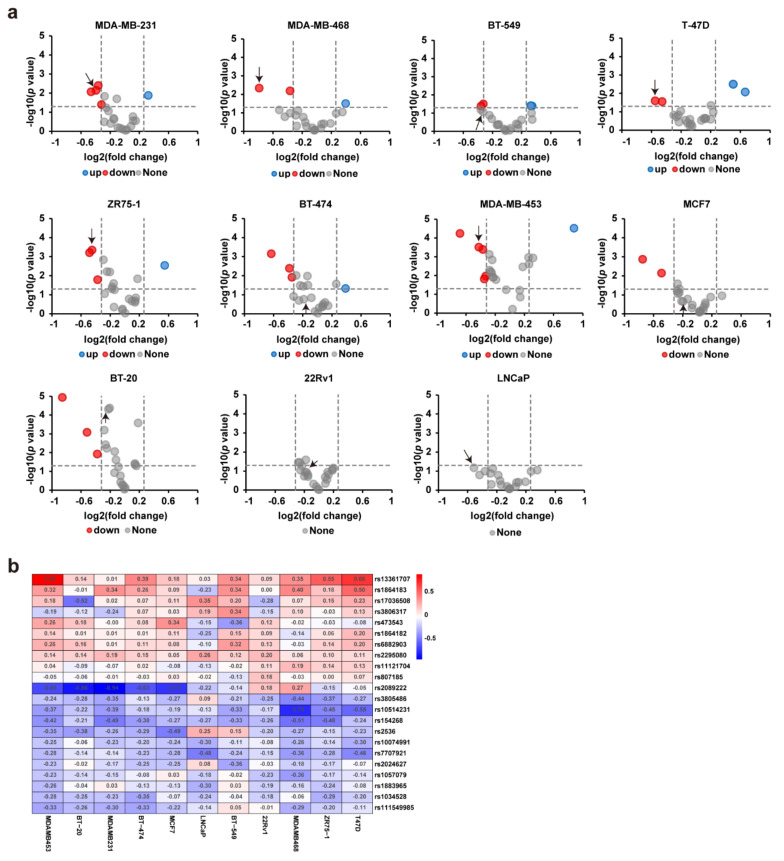
Parallel reporter gene assays identify multiple regulatory SNPs. (**a**) The DiR-seq reporter gene assay for the 22 risk SNPs in nine breast cancer cells and two prostate cancer cells. Red points represent SNP sites with decreased regulatory activity for the risk allele (risk/protective <0.8, *p* < 0.05), and blue points represent SNPs with increased activity for the risk allele (risk/protective >1.2, *p* < 0.05). The *p* values were assessed using a two-tailed Student’s *t*-test. The point highlighted with the arrow is rs10514231. (**b**) Heatmap showing the gene regulation activity of the 22 SNPs in breast cancer cells, annotated values are log2 (risk/protective).

We first performed scatter plotting with the barcode reads in the cDNAs between the biological replicates and evaluated the data’s reliability. The high correlation between the biological replicates indicated good reproducibility between the experiment triplicates (Figure S1). We calculated the regulatory activity fold change for the risk allele over the protective allele to identify the SNPs that possess allele-specific gene regulatory activity. In this step, we discovered multiple regulatory SNP sites for which the risk allele exhibited significantly decreased or increased regulatory activity than the protective allele (Figure 1a). Notably, the DiR reporter expression patterns of the 22 SNP sites exhibited remarkable differences in the 11 cell lines. Among these SNP sites, the rs10514231 site exhibited decreased regulatory activity for the risk allele than the protective allele in most of the nine breast cancer cell lines we used (Figure 1b). Meanwhile, some SNPs, such as the rs2089222 site, showed polarized expression patterns, with a significantly decreased reporter level for the risk allele in the MDA-MB-453, BT-20, BT-474, and MCF7 cell lines, but increased in the MDA-MB-468 cell line to the contrary (Figure 1b).

### 3.2. Rs10514231 Shows Enhancer Activity in Breast Cancer Cells

**Figure 2:**
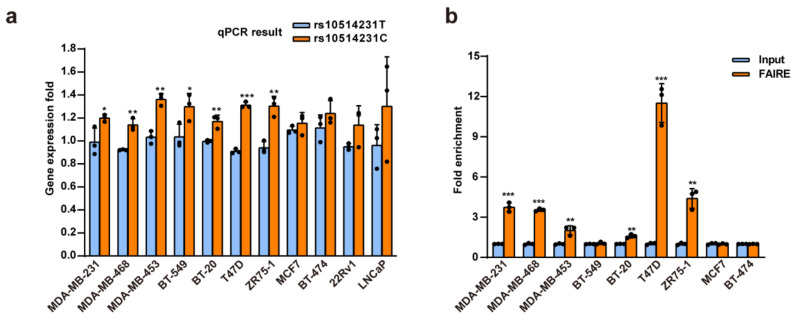
Rs10514231 shows enhancer activity in breast cancer cells. (**a**) The reporter expression level for each allele of rs10514231 was determined via RT-qPCR. MDA-MB-231, MDA-MB-468, MDA-MB-453, BT-549, and BT-20 were classified as triple-negative breast cancer (TNBC) cell lines. T47D, ZR75-1, and MCF7 were LumA cell lines. BT-474 was LumB and also Her2+ cell lines. Values are the means ± SD; * *p* < 0.05, ** *p* < 0.01, and *** *p* < 0.001, two-tailed Student’s *t* test. (**b**) Chromatin open status of the rs10514231 region in breast cancer cells, detected by FAIRE-qPCR. Values are the means ± SD; * *p* < 0.05, ** *p* < 0.01, and *** *p* < 0.001, two-tailed Student’s *t* test.

We then explored the biological function of the rs10514231 alleles systematically. Firstly, we verified the reporter expression level of the two alleles by RT-qPCR (Figure 2a). Consistent with the DiR-seq analysis, the protective C allele showed higher activity than the risk T allele. Active regulatory elements usually have higher chromatin open status on the genome, and hence the chromatin open state has become an important indicator for gene regulation elements [13]. We performed formaldehyde-assisted isolation of the regulatory elements (FAIRE) assay followed by qPCR analysis to determine the open status of the rs10514231 region. The results showed that the rs10514231 site was significantly enriched in the open chromatin fraction in the breast cancer cell lines in which the SNP showed regulatory activity (Figure 2b). This evidence indicates that the rs10514231 locus is an essential gene regulatory element in breast cancer cells.

The rs10514231 is located in the second intron of *ATG10* and has been reported for association with breast cancer risk (C allele: OR = 0.75, 95%CI: 0.59–0.93, *p* = 0.010) [20]. Besides, it also exhibited a significant association with a predisposition for lung cancer, hepatocellular carcinoma, and nasopharyngeal carcinoma [21,22,23]. Nevertheless, the pathogenesis mechanism was poorly investigated. It is of great significance to reveal the biological mechanism through which the variant rs10514231 influences breast cancer risk.

### 3.3. rs10514231 Regulates the Expression of ATP6AP1L

**Figure 3:**
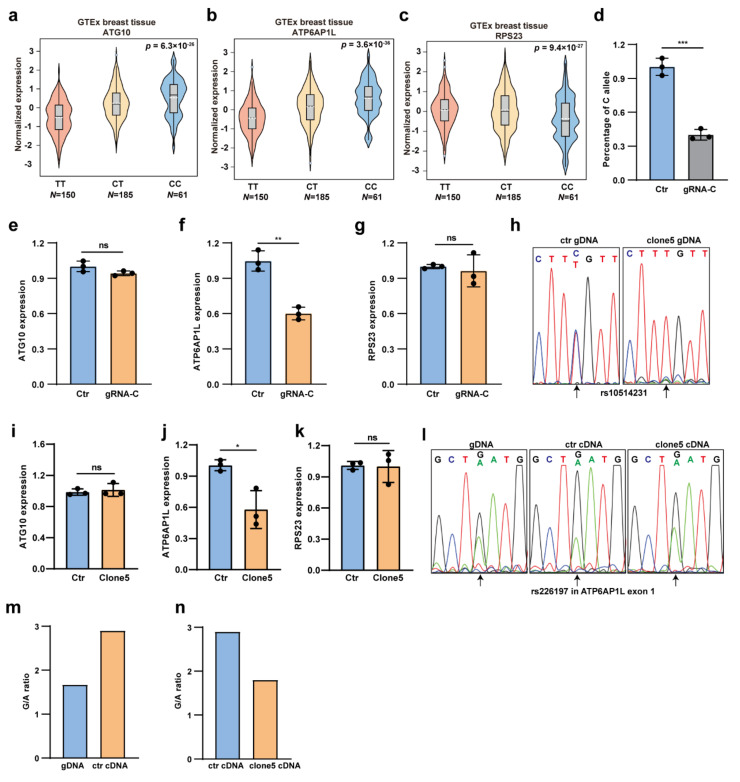
Rs10514231 regulates gene expression of *ATP6AP1L*. (**a**–**c**) The eQTL analysis from the GTEx database showing the association between the rs10514231 genotype and expression of *ATG10*, *ATP6AP1L*, and *RPS23* in normal breast tissues. (**d**) CRISPR/Cas9-mediated C allele-specific genome editing on the rs10514231 site in MDA-MB-453 cells. The remaining level of the C allele was determined by the getPCR method. Values are the means ± SD; * *p* < 0.05, two-tailed Student’s *t*-test. (**e**–**g**) Gene expression level of the three target genes, *ATG10*, *ATP6AP1L*, and *RPS23*, in MDA-MB-453 cells edited with C allele-specific gRNA. Gene expression level determined by RT-qPCR. Values are the means ± SD; ** *p* < 0.01, ns = not significant, two-tailed Student’s **t**-test. (**h**) Sanger sequencing chromatogram of the rs10514231 region in clone 5, a T/T genotype MDA-MB-453 cell line, originated from the C allele-specific genome editing. (**i**–**k**) Gene expression level of the three eQTL genes in the clone 5 cell line, analyzed through RT-qPCR. Values are the means ± SD; * *p* < 0.05, ns = not significant, two-tailed Student’s *t*-test. (**l**) Sanger sequencing chromatogram of rs226197 in *ATP6AP1L* exon 1 in genomic DNA and cDNA of the clone 5 cells and original MDA-MB-453 cells. The altered relative peak height represents the allele-specific expression of the *ATP6AP1L* gene. (**m**,**n**) The under-peak area of the G and A allele in the Sanger sequencing chromatogram quantified by EditR 1.0.9 online software (https://moriaritylab.shinyapps.io/editr_v10/, accessed on 14 October 2020).

We next sought to find the target gene of the rs10514231 regulatory element. The expression quantitative trait locus (eQTL) analysis using data from the GTEx database revealed a strong association between the genotypes of rs10514231 with the expression of *ATG10*, *ATP6AP1L*, and *RPS23* in normal breast tissue (Figure 3a–c). Notably, for both the *ATG10* and *ATP6AP1L* genes, the increased expression was associated with the C allele of rs10514231.

We then performed CRISPR/Cas9-mediated genome editing on the higher activity C allele in heterozygous MDA-MB-453 cells to test the direct regulatory effect of rs10514231 on gene expression. The genome-editing efficiency on the C allele was around 60%, as determined by the getPCR method [16] (Figure 3d). We analyzed the expression level of the three potential eQTL genes through RT-qPCR and found that only *ATP6AP1L* expression was affected by the genome-editing event and had a 40% reduction in the edited cells (Figure 3e–g). We also performed the same genome editing on allele C in the C/C homozygous MDA-MB-468 cells and found that *ATP6AP1L* expression was also downregulated along with the C allele editing (Figure S2a,b). It suggests that the *ATP6AP1L* gene might be the potential target gene of the rs10514231 site. Furthermore, we isolated single-cell clones from the genome-edited MDA-MB-453 cells and established a single-cell clone #5, which was proven to be T/T homozygous at rs10514231 by Sanger sequencing (Figure 3h). In this clone, homology-directed repair (HDR), taking the T allele as a template, might happen after C allele-specific Cas9 cleavage. We further assessed the expression level of the three eQTL genes in this single-cell clone and found that *ATP6AP1L* was the only one affected by the rs10514231 genome editing (Figure 3i–k). Besides, we selected a heterozygous (G/A) SNP, rs226197, in the first exon of *ATP6AP1L* in MDA-MB-453 cells to evaluate the allele-specific expression. We performed Sanger sequencing with the genomic DNA and cDNA surrounding the rs226197 region and found that the expression of rs226197 in the *ATP6AP1L* gene was highly G allele-specific in MDA-MB-453 cells. Notably, when the rs10514231 site shifted from T/C to T/T genotype in clone 5, the allele-preference was impeded obviously (Figure 3l). Interestingly, quantification of the allele ratio in genomic DNA and cDNA samples, by measuring the under-peak area of each allele in the Sanger sequencing chromatogram with EditR 1.0.9 [24], further confirmed the results (Figure 3m,n). Collectively, these results demonstrate that the *ATP6AP1L* gene is supposed to be the direct target of regulatory rs10514231, and the risk T allele can downregulate its expression.

### 3.4. Clinical Impact of ATP6AP1L Expression on Breast Cancer Progression

**Figure 4:**
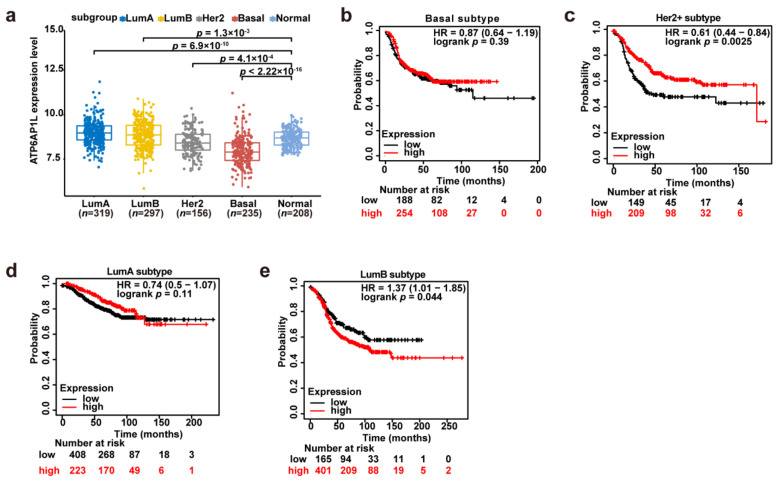
Association between *ATP6AP1L* expression and breast cancer risk. (**a**) The *ATP6AP1L* mRNA expression level was analyzed in different subgroups of breast cancer in TCGA cohorts. The *p* values were examined by Mann–Whitney U tests. (**b**–**e**) Kaplan–Meier plots examining the risk of *ATP6AP1L* for recurrence-free survival in different subtype breast cancer were performed by the Kaplan–Meier plotter online in 2032 BRCA patients. The *p* values were calculated by a log-rank test.

We next investigated the clinical impact of *ATP6AP1L* expression on breast cancer progression. Notably, we found that the *ATP6AP1L* expression level was significantly lower in the basal-like and Her2+ breast cancer samples than in normal samples (Figure 4a). We performed a survival analysis on the different subtypes of breast cancer patients from the Kaplan–Meier plotter database [25] and found that the recurrence-free survival time was longer for patients with higher *ATP6AP1L* expression levels in most subtypes, such as the basal-like, Her2+, and LumA subtypes (Basal: HR = 0.87, 95% CI, 0.64–1.19, log-rank *p* = 0.39; Her2+: HR = 0.61, 95% CI, 0.44–0.84, log-rank *p* = 0.0025; LumA: HR = 0.74, 95% CI, 0.5–1.07, log-rank *p* = 0.11; and LumB: HR = 1.37, 95% CI, 1.01–1.85, log-rank *p* = 0.044) (Figure 4b–e). It suggests that the *ATP6AP1L* gene might function as a suppressor in breast cancer progression, except for the LumB subtype breast cancer.

### 3.5. ATP6AP1L Overexpression Suppresses the Cancerous Phenotype

**Figure 5:**
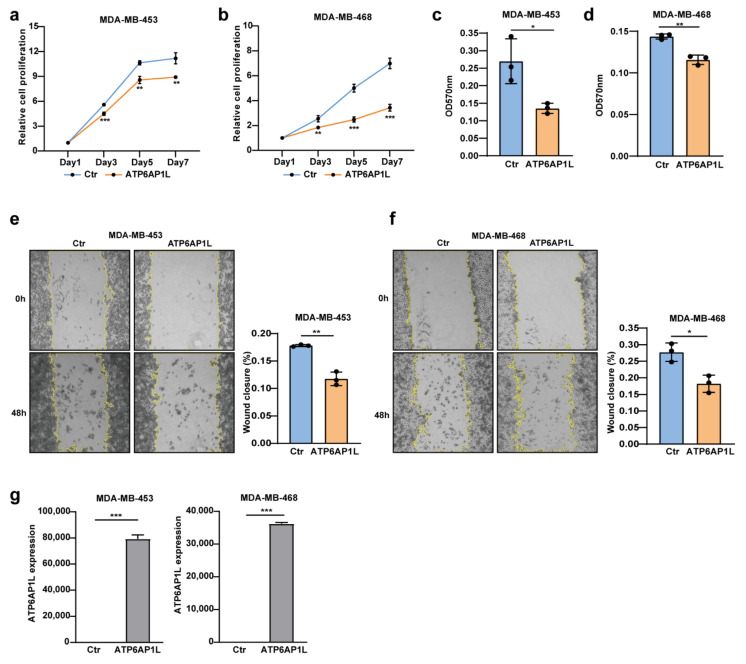
*ATP6AP1L* overexpression suppresses TNBC cell proliferation and migration. (**a**,**b**) Cell proliferation assay of two TNBC cell lines, MDA-MB-453 (**a**) and MDA-MB-468 (**b**), which were overexpressed with the *ATP6AP1L* gene by lentiviral infection. Empty vector packaged virus was used as the control. Cells numbers were measured at the indicated time points with the CCK-8 assay and represented as absorbance at 450 nm. Values are the means ± SD; ** *p* < 0.01, *** *p* < 0.001, two-tailed Student’s *t*-test. (**c**,**d**) Colony-forming assay for MDA-MB-453 (**c**) and MDA-MB-468 (**d**) cells that were overexpressed with the *ATP6AP1L* gene by lentiviral infection. Colonies were stained with crystal violet and quantified by reading absorbance at 570nm. Values are the means ± SD; * *p* < 0.05, ** *p* < 0.01, two-tailed Student’s *t*-test. (**e**,**f**) The wound-healing assay images for MDA-MB-453 (**e**) and MDA-MB-468 (**f**), which were overexpressed with the *ATP6AP1L* gene by lentiviral infection. The histogram on the right depicts the quantification of the wound closure percentage fraction. Values are the means ± SD; * *p* < 0.05, ** *p* < 0.01, two-tailed Student’s *t*-test. (**g**) *ATP6AP1L* overexpression in MDA-MB-453 and MDA-MB-468 cells was analyzed by qPCR. Values are the means ± SD; *** *p* < 0.001, two-tailed Student’s *t*-test.

Since the SNP rs10514231 showed regulatory activity in the TNBC and LumA subtype cell lines, as shown in Figure 2a, we further tested the effect of the *ATP6AP1L* gene on the cancerous genotypes on two TNBC cell lines, MDA-MB-468 and MDA-MB-453, and on the LumA cell line T47D. We found that lentiviral vector-mediated *ATP6AP1L* gene overexpression significantly inhibited cell proliferation for MDA-MB-453, MDA-MB-468, and T47D cells (Figure 5a,b and Figure S3a). The colony-forming assay also showed that *ATP6AP1L* overexpression significantly impeded the cancer cells’ ability to survive and form colonies in MDA-MB-453 and MDA-MB-468 cells (Figure 5c,d), and *ATP6AP1L* overexpression showed the same impact in T47D cells despite no significance (Figure S3b). In addition, cell migration ability is another vital cancerous feature associated with tumor metastasis. Our wound healing assays indicated that lentiviral overexpression of *ATP6AP1L* in MDA-MB-453, MDA-MB-468, and T47D cells significantly suppressed cell migration ability (Figure 5e,f and Figure S3c). The overexpression of the *ATP6AP1L* gene in MDA-MB-453, MDA-MB-468, and T47D cells was confirmed through RT-qPCR analysis, as showed in Figure 5g and Figure S3d.

### 3.6. TCF7L2 Binds the rs10514231 Region and Regulates ATP6AP1L Expression

**Figure 6:**
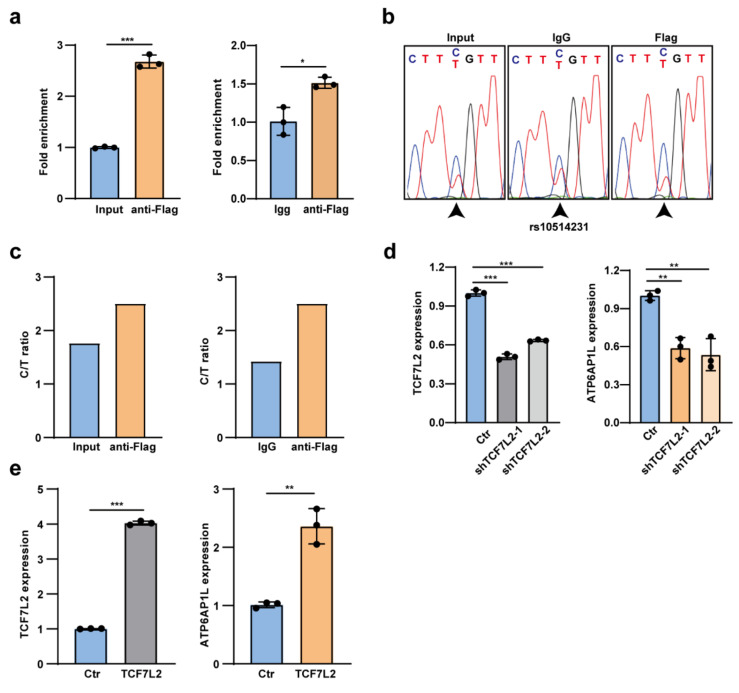
TCF7L2 binds the rs10514231 region and regulates the *ATP6AP1L* gene expression. (**a**) ChIP-qPCR analysis for TCF7L2 chromatin binding at the rs10514231 region. Input DNA and IgG ChIP DNA were used as the negative control. Values are the means ± SD; * *p* < 0.05, *** *p* < 0.001, two-tailed Student’s *t*-test. (**b**) Sanger sequencing chromatogram with the PCR product of ChIP DNA and input DNA. (**c**) The under-peak area of C and T alleles in (**b**) was quantified by EditR 1.0.9 online software. (**d**) RT-qPCR analysis of *ATP6AP1L* expression in MDA-MB-453 cells with lentiviral *TCF7L2* shRNA knockdown. Values are the means ± SD; ** *p* < 0.01, *** *p* < 0.001, two-tailed Student’s *t*-test. (**e**) The mRNA expression level of the *ATP6AP1L* gene in MDA-MB-453 cells upon lentiviral overexpression of the *TCF7L2* gene. Values are the means ± SD; ** *p* < 0.01, *** *p* < 0.001, two-tailed Student’s *t*-test.

It has been widely known that the risk SNPs usually a exert gene regulatory effect through altering the chromatin binding of a given transcription factor [14,26,27]. Hence, it is of great importance to disclose the potential transcription factor that binds the rs10514231 SNP region and regulates the *ATP6AP1L* gene. Our investigation with the online tool HaploRegv4.1 [28] and JASPAR showed that the rs10514231 site might alter the chromatin binding of TCF7L2 (also known as TCF4). To verify this finding, we performed a chromatin immunoprecipitation (ChIP) assay using anti-Flag antibody in MDA-MB-453 cells overexpressed with N-terminal Flag-tagged TCF7L2. The ChIP-qPCR analysis showed that the rs10514231 region was significantly enriched in the TCF7L2 ChIP DNA (Figure 6a).

Interestingly, Sanger sequencing analysis of the PCR product disclosed that TCF7L2 binding preferred the protective C allele of rs10514231, which exhibited higher gene regulatory activity in the reporter assay (Figure 6b,c). Notably, upon knockdown of the *TCF7L2* gene expression in MDA-MB-453 cells, the expression level of *ATP6AP1L* was also significantly impeded (Figure 6d). Meanwhile, the ectopic overexpression of *TCF7L2* dramatically upregulated the *ATP6AP1L* expression (Figure 6e). Briefly, we disclosed that the risk T allele of rs10514231 impeded TCF7L2 binding on the therein chromatin region and led to attenuated *ATP6AP1L* expression.

## 4. Discussion

Even though the role of autophagy in cancer development and progression is complicated, depending on the cell context, tissue type, and expression levels of the core genes [29,30,31], autophagy has become a promising target in breast cancer treatment. It can be pharmacologically modulated through either stimulation or suppression in numerous pathways, and clinical studies have tested the effects of autophagy modulators on breast cancer therapies [32,33]. Hence, elucidating the biological function and underlying mechanism of the autophagy gene-related risk SNPs has great importance in cancer clinical practice.

We evaluated 22 cancer risk SNPs in autophagy-related genes for their gene regulatory activity with the DiR system and discovered multiple functional SNP sites, among which rs10514231 showed significant activity in most breast cancer cells we used. This SNP was located in the second intron of the *ATG10* gene and had been reported to have a significant association with susceptibility for breast cancer and many other diseases in Chinese populations [21]. Analysis using the online tool HaploRegv4.1 revealed that rs10514231 was located in the enhancer histone modification marks in more than ten types of cells [22]. Besides, our FAIRE qPCR also verified that rs10514231 lay in the high open status chromosome region.

For translating the risk SNP information into an improved understanding of the disease susceptibility, it is essential to identify the target genes of the risk-associated variants [34]. Through eQTL analysis with the GTEx database and further gene expression assay on CRISPR/Cas9 genome-edited cells, we identified *ATP6AP1L* to be the target gene for rs10514231. TWAS (transcriptome-wide association study) analysis identified that the *ATP6AP1L* gene was significantly associated with breast cancer risk [35]. It was also reported that the increased expression of *ATP6AP1L* was associated with decreased breast risk [36]. Our study observed attenuated expression of *ATP6AP1L* in the basal-like and Her2+ subtypes of the breast cancer samples compared to normal breast tissues and a shorter recurrence-free survival time with the lower expression group in most subtypes of breast cancer patients. Consistently, our experiments validated that *ATP6AP1L* overexpression inhibited breast cancer cell proliferation and migration, suggesting that *ATP6AP1L* might play a tumor-suppressor role in breast cancer progression. In addition, we revealed that the transcription factor TCF7L2 directly bound the rs10514231 region with a preference for the C allele and mediated the activation of *ATP6AP1L*.

A major remaining question will be how *ATP6AP1L* influenced breast cancer. The product of the *ATP6AP1L* (ATPase H+ Transporting Accessory Protein 1 Like) gene is critical for proton transportation [37]. It was reported that vesicular pH control is essential for cellular signaling (particularly Wnt, Notch, and mTOR signaling) and some cellular processes, especially for autophagy, which was highly dependent on the acidic pH to support the activity of proteases in the lysosome and possibly lysosome/autophagosome fusion [38,39]. Further investigation into the mechanism of *ATP6AP1L* in breast cancer will provide additional insight into the etiology of breast cancer. In-depth studies, such as tumor xenograft experiments, might be demanded to thoroughly investigate the roles of the *ATP6AP1L* gene in breast cancer before being translated into clinics.

## 5. Conclusions

In summary, we disclosed multiple regulatory risk SNPs through a parallel reporter gene assay and elucidated the function and mechanism of rs10514231 in breast cancer susceptibility, which will be valuable for breast cancer risk prediction and effective targeted therapy for cancer patients. A similar procedure could be further applied to help identify and validate functional variants in other diseases.

## Data Availability

The data presented in this study can be made available upon request from the corresponding author.

## Supplementary Materials

The following are available online at https://www.mdpi.com/article/10.3390/cancers13153752/s1, Figure S1: DiR-seq assay in the 11 cell lines, Figure S2: Genome editing on rs10514231 in MDA-MB-468 cell, Figure S3: *ATP6AP1L* overexpression suppresses T47D cell proliferation and migration. Table S1: SNP information:, Table S2: the SNP-centered DNA sequence, Table S3: qPCR primers.
