# Supplementary material for: rs10514231 Leads to Breast Cancer Predisposition by Altering ATP6AP1L Gene Expression
Source: Cancers (Basel). 2021 Jul 26;13(15):3752. doi: 10.3390/cancers13153752 (PMC8345087; doi:10.3390/cancers13153752)
Supplement: Supplementary file 1 [file cancers-13-03752-s001.zip › cancers-1239891-supplementary.pdf]

Article

rs10514231 Leads to Breast Cancer Predisposition by Altering *ATP6AP1L* Gene Expression

Shumin Ma, Naixia Ren and Qilai Huang

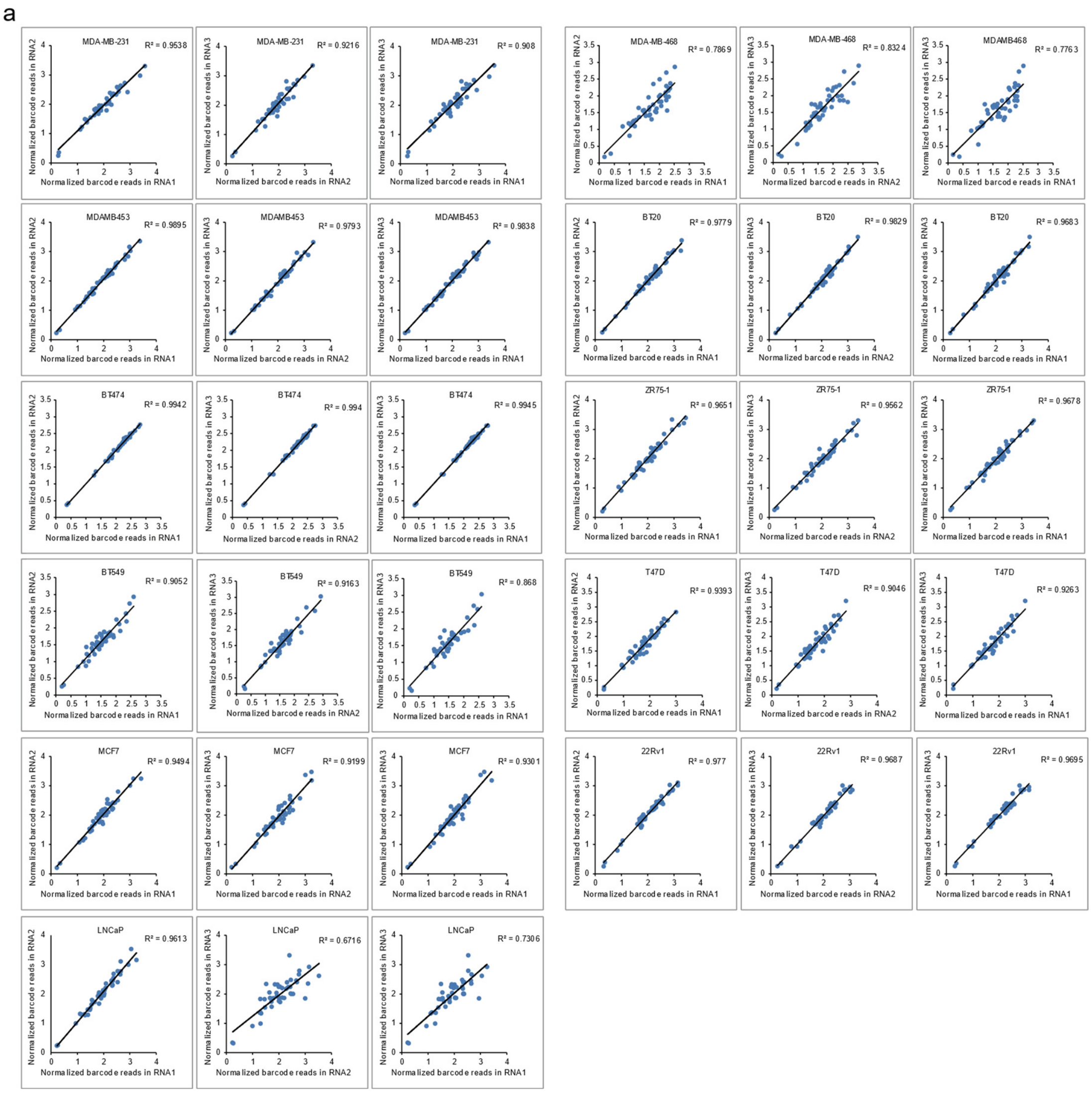

Figure S1. DiR-seq assay in the 11 cell lines. (a) Correlation analysis of barcode counts between each biological replicate for DiR-seq analysis of the 22 SNPs in nine breast cancer cell lines and two prostate cancer cell lines. The black line showed the linear trend, R^2 = Coefficient of determination.

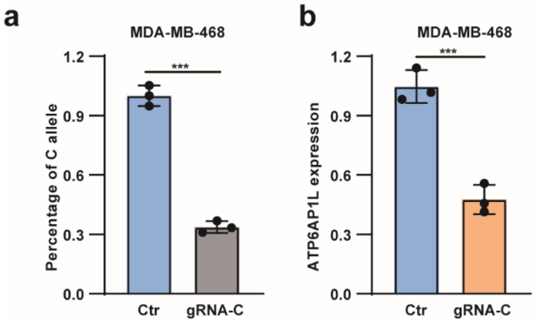

Figure S2. Genome editing on rs10514231 in MDA-MB-468 cell (a) CRISPR/Cas9-mediated C allele-specific genome editing on the rs10514231 site in MDA-MB-468 cell. The remaining level of the C allele was determined by the getPCR method. Values are the means ± SD, ***P<0.001, two-tailed Student's t-test. (b) Gene expression level of *ATP6AP1L* in MDA-MB-468 cell edited with C allele-specific gRNA. Gene expression level determined by RT-qPCR. Values are the means ± SD, ***P<0.001, two-tailed Student's t-test.

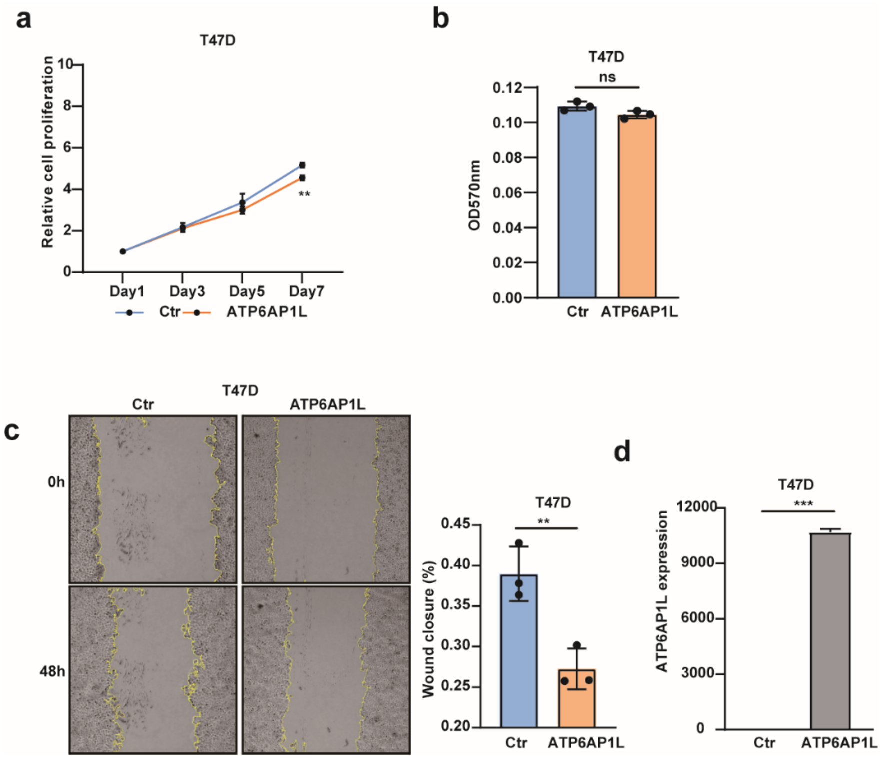

Figure S3. *ATP6AP1L* overexpression suppresses T47D cell proliferation and migration. (a) Cell proliferation assay of T47D cells that were overexpressed with *ATP6AP1L* gene by lentiviral infection. Empty vector packaged virus was used as control. Cells numbers were measured at the indicated time points with CCK-8 assay and represented as absorbance at 450 nm. Values are the means ± SD, **P<0.01, two-tailed Student's t-test. (b) Colony formation assay for T47D cells that were overexpressed with *ATP6AP1L* gene by lentiviral infection. Colonies were stained with crystal violet and quantified by reading absorbance at 570nm. Values are the means ± SD, ns, not significant, two-tailed Student's t-test. (c) The wound-healing assay images for T47D cells that were overexpressed with *ATP6AP1L* gene by lentiviral infection. The histogram on the right was quantified wound closure percentage fraction. Values are the means ± SD, **P<0.01, two-tailed Student's t-test. (d) *ATP6AP1L* overexpression level in T47D cells were analyzed by qPCR. Values are the means ± SD, ***P<0.001, two-tailed Student's t-test.

Table S1.

SNP	Ref allele frequency	Alt allele frequency	OR (95% CI)	Gene	Location	Cancer type
rs1864182	C= 0.0810	A=0.9190,	0.77 (0.61-0.96) [1]	ATG10	exon	breast cancer

		G=0.0000				
rs1864183	C=0.157	T=0.843	0.82 (0.68-1.00) [1]	ATG10	exon	breast cancer
rs10514231	C=0.130	T=0.870	0.75 (0.59-0.93) [1]	ATG10	intron	breast cancer
rs111549985	C=1.000	G=0.000	0.0498 (0.036-0.064) (GWAS)	ATG10	exon	breast cancer
rs7707921	T=0.036	A=0.964	0.94 (0.9-0.98) (GWAS)	ATG10	intron	breast cancer
rs2089222	G=0.7885	A=0.2115	2.26 (1.60-3.0) (GWAS)	ATG8E	intron	leukemia
rs473543	A=0.393	C=0.000, G=0.607	1.73 (1.04-2.87) [2]	ATG5	intron	breast cancer
rs807185	A=0.384	T=0.616	0.605 (0.456-0.803) [3]	ATG4A	intron	lung cancer
rs1034528	G=0.830	C=0.170	1.29 (1.07-1.55) [4]	mTOR	intron	prostate cancer
rs1057079	C=0.188	T=0.812	1.21 (1.05–1.38) [5]	mTOR	intron	colon and rectal cancer
rs11121704	C=0.046	A=0.000, T=0.954	1.53 (1.01-2.32) [6]	mTOR	intron	cancer
rs1883965	A=0.048	G=0.952	1.15 (1.02-1.29) [7]	mTOR	intron	cancer
rs2024627	T=0.040	C=0.960	2.60 (1.16–5.80) [8]	mTOR	intron	cancer
rs2295080	G=0.214	C=0.000, T=0.786	0.77 (0.65-0.92) [9]	mTOR	promoter	gastric cancer
rs2536	T=0.896	C=0.104	1.42 (1.13-1.78) [4]	mTOR	exon	prostate cancer
rs3806317	A=0.912	G=0.088	1.22 (1.00-1.49) [10]	mTOR	intron	gastric cancer
rs17036508	T=0.851	C=0.149	3.73 (1.75-7.94) [11]	mTOR	intron	prostate cancer
rs10074991	G=0.507	A=0.493	1.20 (1.14-1.27) (GWAS)	AMPK	intron	gastric cancer
rs13361707	C=0.5363	T=0.4637	1.41 (1.32-1.49) (GWAS)	AMPK	intron	gastric cancer
rs154268	C=0.267	T=0.733	1.65 (1.22-2.22) [12]	AMPK	intron	gastric cancer
rs6882903	A=0.263	C=0.737	1.48 (1.09-2.00) [12]	AMPK	intron	gastric cancer
rs3805486	A=0.738	G=0.262	0.63 (0.46-0.85) [12]	AMPK	intron	gastric cancer

Table S2.

Primer name.	Sequence(5'--3')
1864182A-F	CAACGTCTCAGGATGAACGAAATGTCCATTAACAAGGTAAAAGAAAAGCCTGGAT
1864182A-R	GATCATCCAGGCTTTTCTTTTACCTTGTTAATGGACATTTCGTTCATCCTGAGACGTTG
1864182C-F	CAACGTCTCAGGATGAACGAAATGTCCCTTAACAAGGTAAAAGAAAAGCCTGGAT
1864182C-R	GATCATCCAGGCTTTTCTTTTACCTTGTTAAGGGACATTTCGTTCATCCTGAGACGTTG
1864183A-F	ATCTACCTCTGAGTTATGCCAAAGCAATGTCTCAGGATGAACGAAATGTCCCTTA
1864183A-R	GATCTAAGGGACATTTCGTTCATCCTGAGACATTGCTTTGGCATAACTCAGAGGTAGAT
1864183G-F	ATCTACCTCTGAGTTATGCCAAAGCAACGTCTCAGGATGAACGAAATGTCCCTTA
1864183G-R	GATCTAAGGGACATTTCGTTCATCCTGAGACGTTGCTTTGGCATAACTCAGAGGTAGAT
10514231A-F	AAAAGATTATTCTTTTGAGCCTGGCTTTGTTCTCTCAACGTTACATTTGTGCATA
10514231A-R	GATCTATGCACAAATGTAACGTTGAGAGAACAAAGCCAGGCTCAAAAGAATAATCTTTT
10514231G-F	AAAAGATTATTCTTTTGAGCCTGGCTTCGTTCTCTCAACGTTACATTTGTGCATA
10514231G-R	GATCTATGCACAAATGTAACGTTGAGAGAACGAAGCCAGGCTCAAAAGAATAATCTTTT
807185A-F	TCTAGTATGGTAACCATAACCACATGTAGCTCTTAAAGACTTGCAATGTGTCTAG
807185A-R	GATCCTAGACACATTGCAAGTCTTTAAGAGCTACATGTGGTTATGGTTACCATACTAGA
807185T-F	TCTAGTATGGTAACCATAACCACATGTTGCTCTTAAAGACTTGCAATGTGTCTAG
807185T-R	GATCCTAGACACATTGCAAGTCTTTAAGAGCAACATGTGGTTATGGTTACCATACTAGA
473543A-F	ACTTCCTGTAATTTTTATAAATTGACCATTTTCTTTCTTTCTCCCCTTGTCCTTC
473543A-R	GATCGAAGGACAAGGGGAGAAAGAAAGAAAATGGTCAATTTATAAAAATTACAGGAAGT
473543G-F	ACTTCCTGTAATTTTTATAAATTGACCGTTTTCTTTCTTTCTCCCCTTGTCCTTC
473543G-R	GATCGAAGGACAAGGGGAGAAAGAAAGAAAACGGTCAATTTATAAAAATTACAGGAAGT
7707921A-F	GATCAGCAAATTTAAGAAATAATCAGATCTTTTAGAAAAGTATATAATGTCTATT
7707921A-R	GATCAATAGACATTATATACTTTTCTAAAAGATCTGATTATTTCTTAAATTTGCTGATC
7707921T-F	GATCAGCAAATTTAAGAAATAATCAGAACTTTTAGAAAAGTATATAATGTCTATT
7707921T-R	GATCAATAGACATTATATACTTTTCTAAAAGTTCTGATTATTTCTTAAATTTGCTGATC
111549985C-F	CTCTCCCCAGCTCTCCTCCCCCTGGCCCCGTCGCCCCGCCCTCGCCGGGCTGGGC
111549985C-R	GATCGCCCAGCCCGGCGAGGGCGGGGCGACGGGGCCAGGGGGAGGAGAGCTGGGGAGAG
111549985G-F	CTCTCCCCAGCTCTCCTCCCCCTGGCCGCGTCGCCCCGCCCTCGCCGGGCTGGGC
111549985G-R	GATCGCCCAGCCCGGCGAGGGCGGGGCGACGCGGCCAGGGGGAGGAGAGCTGGGGAGAG
2295080G-F	CCGGGAGCGAGGGAAGGAGGGTTCCCAGCCCTGAGGACCAATCGACAGGTATAGG
2295080G-R	GATCCCTATACCTGTCGATTGGTCCTCAGGGCTGGGAACCCTCCTTCCCTCGCTCCCGG
2295080T-F	CCGGGAGCGAGGGAAGGAGGGTTCCCATCCCTGAGGACCAATCGACAGGTATAGG
2295080T-R	GATCCCTATACCTGTCGATTGGTCCTCAGGGATGGGAACCCTCCTTCCCTCGCTCCCGG
11121704C-F	TACCTACTTCTCCTTTCCAAACATCTGCGATGATGTGCCTGAAGCATTAACTCTT
11121704C-R	GATCAAGAGTTAATGCTTCAGGCACATCATCGCAGATGTTTGGAAAGGAGAAGTAGGTA
11121704T-F	TACCTACTTCTCCTTTCCAAACATCTGTGATGATGTGCCTGAAGCATTAACTCTT
11121704T-R	GATCAAGAGTTAATGCTTCAGGCACATCATCACAGATGTTTGGAAAGGAGAAGTAGGTA
2536T-F	AGTTGAGTATTTGTTCTGCTCATAATTTCCAATATGTACCAGACCTTCCCTGTGT
2536T-R	GATCACACAGGGAAGGTCTGGTACATATTGGAAATTATGAGCAGAACAAATACTCAACT
2536C-F	AGTTGAGTATTTGTTCTGCTCATAATTCCCAATATGTACCAGACCTTCCCTGTGT
2536C-R	GATCACACAGGGAAGGTCTGGTACATATTGGGAATTATGAGCAGAACAAATACTCAACT
1057079C-F	TGCCCGACTGTAACTCTCTCCTGCCATCGCAGTTAATTCAGCATCCAGCAGGTCC

1057079C-R	GATCGGACCTGCTGGATGCTGAATTAACTGCGATGGCAGGAGAGAGTTACAGTCGGGCA
1057079G-F	TGCCCGACTGTAACTCTCTCCTGCCATGGCAGTTAATTCAGCATCCAGCAGGTCC
1057079G-R	GATCGGACCTGCTGGATGCTGAATTAACTGCCATGGCAGGAGAGAGTTACAGTCGGGCA
2024627T-F	AGGCTACTAATTCTACTTTCAACTAGATATTGATCAATACGATACTGACCCGGAA
2024627T-R	GATCTTCCGGGTCAGTATCGTATTGATCAATATCTAGTTGAAAGTAGAATTAGTAGCCT
2024627C-F	AGGCTACTAATTCTACTTTCAACTAGACATTGATCAATACGATACTGACCCGGAA
2024627C-R	GATCTTCCGGGTCAGTATCGTATTGATCAATGTCTAGTTGAAAGTAGAATTAGTAGCCT
1883965A-F	GGAGGACAAGGGGCTGACACGGACGCTAGTGTTCCCTGGGGCAGAGATACACAAT
1883965A-R	GATCATTGTGTATCTCTGCCCCAGGGAACACTAGCGTCCGTGTCAGCCCCTTGTCCTCC
1883965G-F	GGAGGACAAGGGGCTGACACGGACGCTGGTGTTCCCTGGGGCAGAGATACACAAT
1883965G-R	GATCATTGTGTATCTCTGCCCCAGGGAACACCAGCGTCCGTGTCAGCCCCTTGTCCTCC
1034528G-F	TCTGACCCTGTCCTGCTTGCTGATAGAGTTGAGCCAGTGGCTGGCAGGGGCATAA
1034528G-R	GATCTTATGCCCCTGCCAGCCACTGGCTCAACTCTATCAGCAAGCAGGACAGGGTCAGA
1034528C-F	TCTGACCCTGTCCTGCTTGCTGATAGACTTGAGCCAGTGGCTGGCAGGGGCATAA
1034528C-R	GATCTTATGCCCCTGCCAGCCACTGGCTCAAGTCTATCAGCAAGCAGGACAGGGTCAGA
17036508T-F	TTCCTTCTACAATAAACACTTTCAAAATGTGAGTATCTTCTTTTTAAATTCTTAC
17036508T-R	GATCGTAAGAATTTAAAAAGAAGATACTCACATTTTGAAAGTGTTTATTGTAGAAGGAA
17036508C-F	TTCCTTCTACAATAAACACTTTCAAAACGTGAGTATCTTCTTTTTAAATTCTTAC
17036508C-R	GATCGTAAGAATTTAAAAAGAAGATACTCACGTTTTGAAAGTGTTTATTGTAGAAGGAA
3806317A-F	GACTACAACAGTACAAGTAACTGCCTGAGTGGCTCCATAACATGGAAACAGCAAT
3806317A-R	GATCATTGCTGTTTCCATGTTATGGAGCCACTCAGGCAGTTACTTGTACTGTTGTAGTC
3806317G-F	GACTACAACAGTACAAGTAACTGCCTGGGTGGCTCCATAACATGGAAACAGCAAT
3806317G-R	GATCATTGCTGTTTCCATGTTATGGAGCCACCCAGGCAGTTACTTGTACTGTTGTAGTC
13361707C-F	AGGGCATTCCAAATACCCATGAGCCACCATCAGCTTAAGCAATAAAACATTACAA
13361707C-R	GATCTTGTAATGTTTTATTGCTTAAGCTGATGGTGGCTCATGGGTATTTGGAATGCCCT
13361707T-F	AGGGCATTCCAAATACCCATGAGCCACTATCAGCTTAAGCAATAAAACATTACAA
13361707T-R	GATCTTGTAATGTTTTATTGCTTAAGCTGATAGTGGCTCATGGGTATTTGGAATGCCCT
154268C-F	ATTCTGATCAACATCAACAAATGTGATCATGAACAAATTTAACCATATCTATGAA
154368C-R	GATCTTCATAGATATGGTTAAATTTGTTCATGATCACATTTGTTGATGTTGATCAGAAT
154268T-F	ATTCTGATCAACATCAACAAATGTGATTATGAACAAATTTAACCATATCTATGAA
154268T-R	GATCTTCATAGATATGGTTAAATTTGTTCATAATCACATTTGTTGATGTTGATCAGAAT
6882903A-F	AAATGAAGGAATTGAGGTTAAAAAAAAAATCAAGGAACATGCTCAAGGTCACAAA
6882903A-R	GATCTTTGTGACCTTGAGCATGTTCCTTGATTTTTTTTTTAACCTCAATTCCTTCATTT
6882903C-F	AAATGAAGGAATTGAGGTTAAAAAAAAACATCAAGGAACATGCTCAAGGTCACAAA
6882903C-R	GATCTTTGTGACCTTGAGCATGTTCCTTGATGTTTTTTTTTAACCTCAATTCCTTCATTT
10074991G-F	GATAGGGCTTTTTGCTCCCAGCCTATAGTGTTCCAGCTCGTGTGGTTTTCCGCTT
10074991G-R	GATCAAGCGGAAAACCACACGAGCTGGAACACTATAGGCTGGGAGCAAAAAGCCCTATC
10074991A-F	GATAGGGCTTTTTGCTCCCAGCCTATAATGTTCCAGCTCGTGTGGTTTTCCGCTT
10074991A-R	GATCAAGCGGAAAACCACACGAGCTGGAACATTATAGGCTGGGAGCAAAAAGCCCTATC
3805486T-F	GTCACTGGAGACATTCGTGTCACAGCCATATCACAGATCAAATCCAGAAGATGAA
3805486T-R	GATCTTCATCTTCTGGATTTGATCTGTGATATGGCTGTGACACGAATGTCTCCAGTGAC
3805486C-F	GTCACTGGAGACATTCGTGTCACAGCCGTATCACAGATCAAATCCAGAAGATGAA
3805486C-R	GATCTTCATCTTCTGGATTTGATCTGTGATACGGCTGTGACACGAATGTCTCCAGTGAC
2089222A-F	GCATGGGATCCTCCTCCCTGTGCTGCAATCTAAAAAGCTCTTCCAGGCAGAAAGCA
2089222A-R	GATCTGCTTTCTGCCTGGAAGAGCTTTTTAGATTGCAGCACAGGGAGGAGGATCCCATGC
2089222G-F	GCATGGGATCCTCCTCCCTGTGCTGCAGTCTAAAAAGCTCTTCCAGGCAGAAAGCA
2089222G-R	GATCTGCTTTCTGCCTGGAAGAGCTTTTTAGACTGCAGCACAGGGAGGAGGATCCCATGC

Table S3.

Primer name	Experiment	Sequence(5'--3')
ATG10-94f	qPCR test for ATG10	AGACCATCAAAGGACTGTTCTGA
ATG10-94r	qPCR test for ATG10	GGGTAGATGCTCCTAGATGTGAC
TCF7L2-87RT-f	qPCR test for TCF7L2	TGGAGGGCTCTTTAAGGGG
TCF7L2-87RT-r	qPCR test for TCF7L2	GATCCGTTGGGGAGGTAGG
ATP6AP1L-107f	qPCR test for ATP6AP1L	ACTGGCGTATATGCTCCCTCT
ATP6AP1L-107r	qPCR test for ATP6AP1L	CTCGACCCATCATCCGTGTC
RPS23-144f	qPCR test for RPS23	GGTGCTTCTCATGCAAAAGGA
RPS23-144r	qPCR test for RPS23	GCAACCGTCATTGGGTACAAA
10514231-109F	FAIRE or ChIP qPCR for rs10514231	GCACGCAAAACTCTTGCTTA
10514231-109R	FAIRE or ChIP qPCR for rs10514231	GCATAACCTCTGACCAATCATC
ChIPNeg135-f	ChIP-qPCR for control	TGCCTCAGATTTGGAGTGCT
ChIPNeg135-r	ChIP-qPCR for control	GAGAAGCCTCTGAGGAGGGA
10514231Ctrl-82f	getPCR for control	CTGCGTCTACACAAAGCCTCCTCA
10514231Ctrl-82r	getPCR for control	CCACTCACATGTGTAGGGGAAGCT
10514231T1Co4-f-2	getPCR for C allele of rs10514231	AAGATTATTCTTTTGAGCCTGGCTTCG
10514231-113r	getPCR for C allele of rs10514231	AGCCTACTGGCTCCAGGTGTTTG

References:

1. Qin, Z.; et al., *Potentially functional polymorphisms in ATG10 are associated with risk of breast cancer in a Chinese population.* Gene, 2013. **527**(2): P. 491-5.
2. Li, M.; et al., *Genetic polymorphisms of autophagy-related gene 5 (ATG5) rs473543 predict different disease-free survivals of triple-negative breast cancer patients receiving anthracycline- and/or taxane-based adjuvant chemotherapy.* Chin J Cancer, 2018. **37**(1): P. 4.
3. He, Q.; et al., *An. intron SNP rs807185 in ATG4A decreases the risk of lung cancer in a southwest Chinese population.* Eur J Cancer Prev, 2016. **25**(4): P. 255-8.
4. Li, Q.; et al., *Polymorphisms in the mTOR gene and risk of sporadic prostate cancer in an Eastern Chinese population. PLoS ONE,* 2013. **8**(8): P. e71968.
5. Slattery, M.L.; et al., *Genetic variation in a metabolic signaling pathway and colon and rectal cancer risk: mTOR, PTEN, STK11, RPKAA1, PRKAG2, TSC1, TSC2, PI3K and Akt1.* Carcinogenesis, 2010. **31**(9): P. 1604-11.
6. Shao, J.; et al., *Association of mTOR polymorphisms with cancer risk and clinical outcomes: A meta-analysis. PLoS ONE,* 2014. **9**(5): P. e97085.
7. Zining, J.; et al., *Genetic polymorphisms of mTOR and cancer risk: A systematic review and updated meta-analysis.* Oncotarget, 2016. **7**(35): P. 57464-57480.
8. Bonnet, S.; et al., *Effect of genetic polymorphisms in CYP3A4, CYP3A5, and m-TOR on everolimus blood exposure and clinical outcomes in cancer patients.* Pharmacogenomics J, 2020. **20**(5): P. 647-654.
9. Xu, M.; et al., *A polymorphism (rs2295080) in mTOR promoter region and its association with gastric cancer in a Chinese population. PLoS ONE,* 2013. **8**(3): P. e60080.
10. Wang, M.Y.; et al., *Genetic variations in the mTOR gene contribute toward gastric adenocarcinoma susceptibility in an Eastern Chinese population.* Pharmacogenet Genomics, 2015. **25**(11): P. 521-30.
11. Liu, T.; et al., *Gene polymorphisms in the PI3K/AKT/mTOR signaling pathway contribute to prostate cancer susceptibility in Chinese men.* Oncotarget, 2017. **8**(37): P. 61305-61317.
12. Kim, Y.D.; et al., *Risk of gastric cancer is associated with PRKAA1 gene polymorphisms in Koreans.* World J Gastroenterol, 2014. **20**(26): P. 8592-8.

.
